# A Systematical Survey on the TRP Channels Provides New Insight into Its Functional Diversity in Zhikong Scallop (*Chlamys farreri*)

**DOI:** 10.3390/ijms222011075

**Published:** 2021-10-14

**Authors:** Cheng Peng, Zujing Yang, Zhi Liu, Shenhai Wang, Haitao Yu, Chang Cui, Yuqing Hu, Qiang Xing, Jingjie Hu, Xiaoting Huang, Zhenmin Bao

**Affiliations:** 1 MOE Key Laboratory of Marine Genetics and Breeding, College of Marine Life Sciences, Ocean University of China, Qingdao 266000, China; pengcmiles@163.com (C.P.); yzj102553@163.com (Z.Y.); liuzhiouc@163.com (Z.L.); haizhixionghuai@sina.com (S.W.); haitao0532@foxmail.com (H.Y.); 17852029717@139.com (C.C.); cnqdhyq@126.com (Y.H.); qiangxing@ouc.edu.cn (Q.X.); hujingjie@ouc.edu.cn (J.H.); zmbao@ouc.edu.cn (Z.B.); 2 Laboratory for Marine Fisheries Science and Food Production Processes, Pilot Qingdao National Laboratory for Marine Science and Technology, Qingdao 266000, China; 3 Laboratory of Tropical Marine Germplasm Resources and Breeding Engineering, SANYA Oceanographic Institution of the Ocean University of CHINA (SOI-OUC), Sanya 572000, China

**Keywords:** TRP channel, *Chlamys farreri*, spatiotemporal expression, heat stress, thermoTRP

## Abstract

Transient receptor potential (TRP) channel plays a significant role in mediating various sensory physiological functions. It is widely present in the vertebrate and invertebrate genomes and can be activated by multiple compounds, messenger molecules, temperature, and mechanical stimulation. Mollusks are the second largest phylum of the animal kingdom and are sensitive to environmental factors. However, the molecular underpinnings through which mollusks sense and respond to environmental stimulus are unknown. In this study, we systematically identified and characterized 17 TRP channels (C.FA TRPs, seven subfamilies) in the genome of the Zhikong scallop (*Chlamys farreri*). All *C.FA* TRPs had six transmembrane structures (TM1–TM6). The sequences and structural features of *C.FA* TRPs are highly conserved with TRP channels of other species. Spatiotemporal expression profiling suggested that some *C.FA* TRPs participated in the early embryonic development of scallops and the sensory process of adult tissues. Notably, the expression of *C.FA* TRPM3 continuously increased during developmental stages and was highest among all *C.FA* TRPs. *C.FA* TRPC-α was specifically expressed in eyes, which may be involved in light transmission of scallop eyes. Under high temperature stress, *C.FA* TRPA1 and *C.FA* TRPA1-homolog upregulated significantly, which indicated that the TRPA subfamily is the thermoTRPs channel of scallops. Our results provided the first systematic study of TRP channels in scallops, and the findings will provide a valuable resource for a better understanding of TRP evolution and function in mollusks.

## 1. Introduction

Transient receptor potential (TRP) channel is an important cation channel in response to various extracellular and intracellular stimuli, including temperature, osmotic pressure, taste, vision, hearing, mechanical sensation, and sensory perception of multiple compounds [1,2,3,4]. TRP channel was first discovered in the light sensory system of *Drosophila melanogaster* in 1960s [5,6,7]. Since then, much more TRP channels were identified in a great variety of multicellular organisms, such as 13 TRP channels in *D. melanogaster* [8], 27 TRP channels in *Ciona intestinalis* [9], 27 TRP channels in *Danio rerio* [10], 28 TRP channels in *Mus musculus*, 27 TRP channels in *Homo sapiens* [11], and 17 TRP channels in *Caenorhabditis elegans* [12] (Table 1). In addition, a vacuolar membrane protein homologous to the TRP channel named Yvc1p (Yeast vacuolar conductance 1 protein) were reported in *Saccharomyces cerevisiae*, which indicated that the origin of TRP channels predated the emergence of metazoan organisms [11]. However, there was still not any TRP homologues reported in land plants although a large number of genomes have been completed. Only in algae *Chlamvdomonas reinhardtii,* TRPL were identified which was homologous with mammalian TRPC channels [13].

According to the amino acid sequences and topological structures, TRP channels are divided into seven subfamilies, including TRPC (Canonical), TRPM (Melastatin), TRPV (Vanilloid), TRPA (Ankyrin), TRPN (Nompc), TRPML (Mucolipin) and TRPP (Polycystin) [14]. The TRPP subfamily is ancient because members of this subfamily extend from yeast to mammals [15]. During the evolution of the TRP family, the gene expansion of the five subfamilies TRPC, TRPM, TRPV, TRPP and TRPML is obvious in vertebrates (Table 1). For example, the TRPC subfamily contains six and seven members, respectively, in *H. sapiens* and *M. musculus*, while only three members are found in *D. melanogaste* and *C. elegans*. However, the number of TRPA subfamilies in invertebrates is more than that in vertebrates. In addition, TRPN proteins are not found in mammals, including *H. sapiens* and *M. musculus*, and TRPC2 is proved to be a pseudogene in *H. sapiens* [16].

Members of the TRP channels share the common features of six transmembrane domains (TM1–TM6), including the pore loop situated between the fifth and sixth transmembrane segments [17]. The N-terminal of TRP family proteins is located in the cell which often contains varying numbers of Ankyrin Repeats (ANKs). For example, the N-terminal of TRPC and TRPV contains 2–6 ANKs, the N-terminal of TRPA contains 14 ANKs, and the N-terminal of TRPN contains 29 ANKs. Each ANKs consists of 33 amino acids and forms a conserved helix-turn-helix structure [18]. The C-terminus of the TRP channel is also located inside the cell, and there are great differences among members of each TRP subfamily. The downstream of the C-terminus of the TRP channel contains a variety of different binding sites, such as the CaM (Calmodulin) binding site, IP3 receptor binding site, PKA/PKC (Protein Kinase A/C) regulatory site and PDZ (Postsynaptic Density Zone) binding site [19]. The TRPP and TRPML proteins share sequence homology over the transmembrane segments and contain a large loop separating the first two transmembrane domains (TM1 and TM2). TRPC, TRPM, and TRPN channels contain a TRP domain, which follows the sixth transmembrane domain [20]. The TRP domain includes 23–25 amino acids and mostly starts with TRP box 1 “EWKFAR” and ends with TRP box 2 “LPPPFN”. TRP box 1 is conserved in TRPCs and varies in TRPNs and TRPMs. TRP box 2 is a region rich in proline [11].

Different types of TRP channels have different activation conditions (such as temperature, vision, hearing, etc.) with different functions including inflammation regulation, cardiovascular regulation, smooth muscle contraction, Ca^2+^ homeostasis, lysosomal function, cell growth and apoptosis [1,21,22,23,24,25]. The interaction of ANKs, transmembrane domains and various regulatory sites among different TRP subfamilies contribute to the function diversity of TRP channels. For example, TRPM8 generally could be activated by cold stimulation and menthol, which is also involved in spermatozoal acrosomal reaction of mouse [26,27] and development of human melanoma [28]. TRPM6 and TRPM7 have high selectivity and permeability of Mg^2+^, and play important roles in maintaining the homeostasis of Mg^2+^ in the kidney and intestine, as well as in the regulation of cell cycle [29,30]. Moreover, the C-terminals of TRPM2, TRPM6 and TRPM7 channels have an enzymatic functional structure, so they are also called “channel enzymes” (such as ADP (Adenosine Diphosphate)—ribose pyrophosphatase and PLC (Phospholipase C)—interacting kinase) [20,29,31]. The TRPM-gon2 is proved to anticipate gonad precursor cell division in *C. elegans* [32]. In addition, some TRP channels activated by temperature are called thermoTRPs (thermosensitive transient receptor potential), which is another good example that shows the diversity function of TRP channels. In mammals, thermoTRPs are divided into thermally activated TRPV (TRPV1, TRPV2, TRPV3, TRPV4) and TRPM (TRPM2, TRM3, TRPM4, TRPM5) [33], and cold activated TRPM8 [34] and TRPA1 [35,36]. The most classic thermoTRP is TRPV1, which can be directly activated by high temperature ≥43 °C in humans [37]. However, the thermoTRPs in invertebrates are completely different from mammals. For example, in *D. melanogaster*, there are at least three TRP channels participating in perception and avoiding of high temperature, which are TRPA-Pyx (Pyrexia), TRPA-Pain (Painless) and TRPA1 [38,39]. All these findings indicate the important roles of TRP channels to different environmental stimulus. However, previous studies on the TRP family mainly focused on mammals, such as *H. sapiens* and *M. musculus*, and invertebrates, such as *D. melanogaster* and *C. elegans*. There are few reports on the TRP family in mollusks, which is the second largest phylum of the animal kingdom and plays important roles in aquatic and marine ecosystems.

The Zhikong scallop (C. farreri, [40]) is a mollusk that is naturally distributed along the coasts of Northern China, Korea, Japan, and Eastern Russia. Previous research indicated that scallops are sensitive to environmental factors such as pH, temperature, and salinity [41,42,43,44]. The growth, development, reproduction, and other life activities of scallops are closely related to the change of environment [45]. However, the molecular mechanism of how scallops adapt to a diverse environment remains unclear. In this study, to gain a better understanding of the diversity functions of TRP channels in mollusks, TRP channels of *C. farreri* were identified and systematically characterized. The spatiotemporal expression profiles of *C.FA TRP* genes were then analyzed using RNA-seq datasets. Furthermore, the expression levels of *C.FA* *TRP* genes in hemolymph, heart, mantle and gill were examined under heat stress to recognize the thermoTRP of scallop.

## 2. Results

### 2.1. Identification and Characterization of TRP Genes in C. farreri

After scanning in both transcriptome and genome database of *C. farreri*, a total of 17 *TRP* genes were identified. The sequence characteristics of *C.FA TRP* genes are summarized in Table 2. Based on domain characteristics and sequence homology, 17 *C.FA TRP* genes were divided into seven TRP subfamilies and were separately named as *C.FA TRPC-α, C.FA TRPC-α1, C.FA TRPC-γ, C.FA TRPC-like, C.FA TRPM2a, C.FA TRPM2b, C.FA TRPM1, C.FA TRPM3, C.FA TRPA1, C.FA TRPA1-homolog, C.FA TRPN, C.FA TRPV-Nan, C.FA TRPV1, C.FA TRPV-Lav1, C.FA TRPV-Lav2, C.FA TRPP,* and *C.FA TRPML*. The open reading frames (ORF) of *C.FA TRP* genes varied from 1614 to 5052 and encoded 537 to 1683 amino acids. The longest *C.FA TRP* gene is *C.FA TRPM3*, and the shortest *C.FA TRP* gene is *C.FA TRPML*. *C.FA TRPM2b* has the largest number of exons among all *C.FA TRP* genes, which was composed of 34 exons and 33 introns. *C.FA TRPV-Lav2* contains only one exon. The predicted molecular weights of the *C.FA TRP* genes ranged from 60.54 to 191.64 kDa, with the predicted isoelectric points (pI) from 5.42 to 8.86. The deduced secondary structures of the protein encoded by *C.FA TRP* genes indicated that these proteins consisted of 22 to 83 alpha helixes, 32 to 87 beta strands, 27 to 119 coils, and 35 to 116 turns. The amino acid identity were 21.70–79.95% between *C.FA* TRPs with TRPs of other invertebrates, and 19.12–57.13% between *C.FA* TRPs with TRPs of vertebrates. The highest identity was 79.95% which occurred in TRPN between scallop and oyster (Table 3).

**Figure 1:**
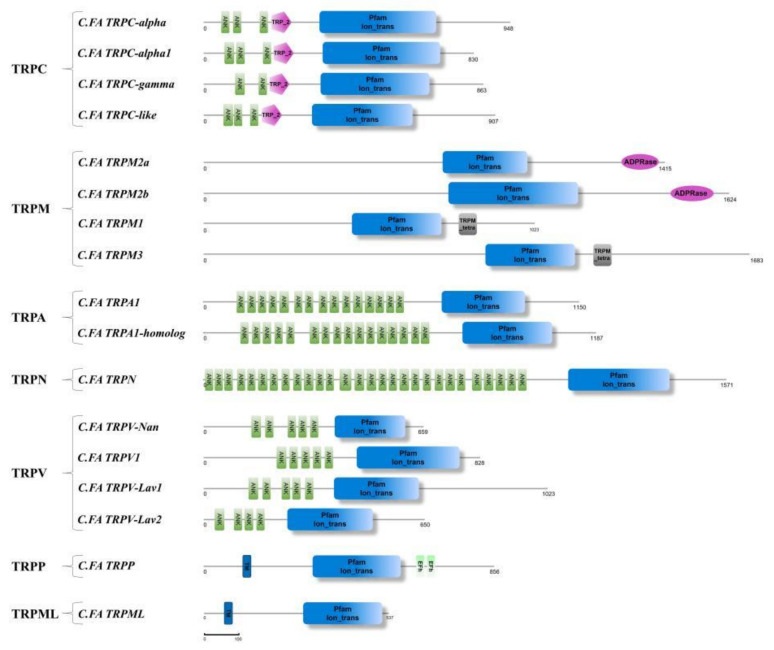
Protein structure of TRP family genes in *C. farreri*. The blue boxes are Pfam Ion trans domain which contains six transmembrane regions. The green boxes are Ankyrin repeat domain. The pink pentagons are the TRP 2 domain. The gray boxes are the TRPM tetra domain. The pink ovals are the ADPRanse domain. The fluorescent green boxes are the EFh domain.

**Figure 2:**
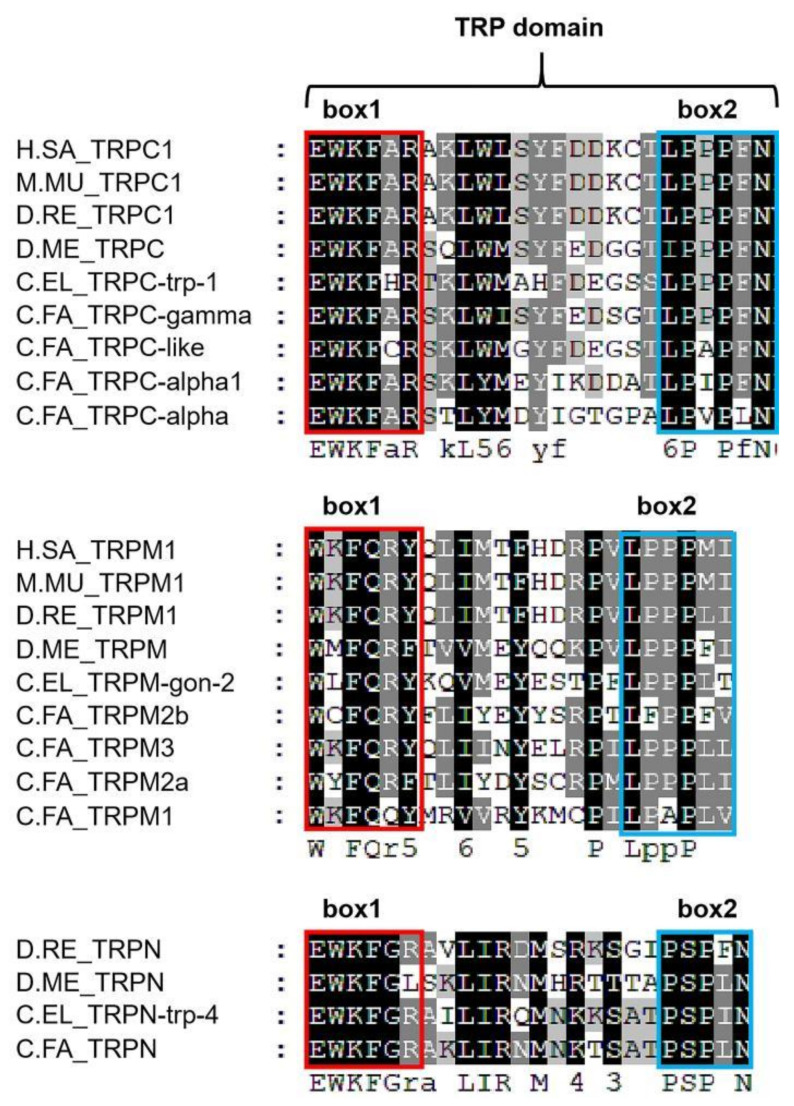
Multi-sequence comparison of the TRP domain in TRPC, TRPM and TRPN. Box 1 frames with red and box 2 frames with blue. H.SA, *H. sapiens*; M.MU, *M. musculus*; D.RE, *D. rerio*; D.ME, *D. melanogaster*; C.EL, *C. elegans*; *C.FA*, *C. farreri*.

Domain analysis showed that there were Pfam Ion trans domains in the middle of all *C.FA* TRPs including six transmembrane domains (TM1–TM6) (Figure 1). There were different numbers of Ankyrin Repeats (ANK) domains in the N-terminus of *C.FA* TRPC, *C.FA* TRPA, *C.FA* TRPN and *C.FA* TRPV subfamilies. There were also some specific structural regions in some *C.FA* TRPs, such as ADPRanse in the C-terminus of *C.FA* TRPM2a and *C.FA* TRPM2b. There was a highly conserved TRP domain behind the TM6 of the *C.FA* TRPC, *C.FA* TRPM and *C.FA* TRPN subfamilies. Multiple alignments of TRPC, TRPM and TRPN subfamilies revealed that the TRP domain exhibits two conserved motifs: box 1 and box 2 (Figure 2). Compared with *H. sapiens*, *M. musculus*, *D. rerio* and *D. melanogaster*, TRP box 1 and TRP box 2 showed variation in TRPCs, TRPNs and TRPMs of scallops. TRP box 2 is a proline-rich (P) region.

### 2.2. Phylogenetic Analysis and Interspecies Comparison of TRP Proteins

**Figure 3:**
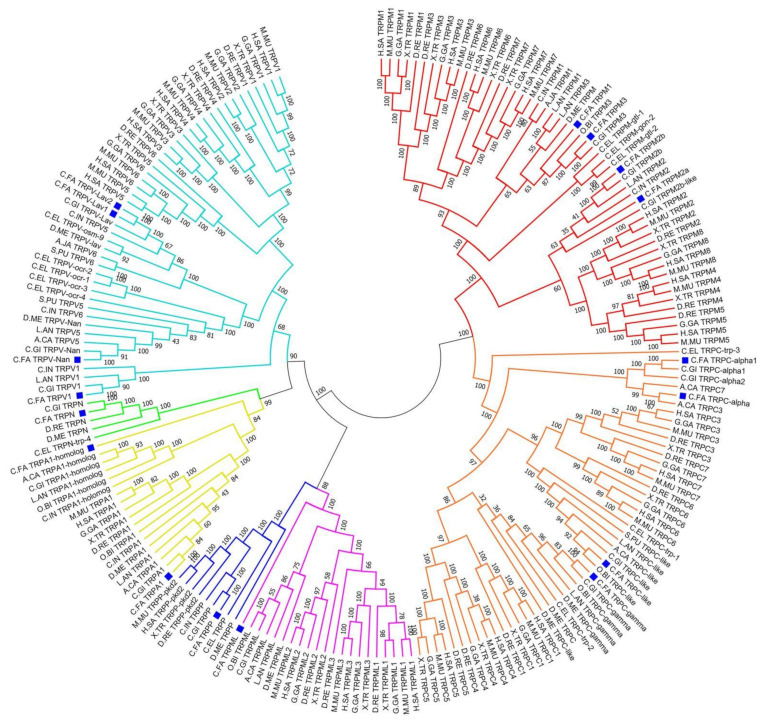
A phylogenetic tree was constructed based on the protein sequences of TRP from *C. farreri* and other selected species using neighbor-joining method with bootstrapping of 1000 pseudo replicates. *C.FA* TRPs are marked with blue squares. Branches of different subfamily are highlighted with different color. (TRPM subfamily: red, TRPC subfamily: orange, TRPA subfamily: yellow. TRPN subfamily: green; TRPV subfamily: light blue; TRPP subfamily: dark blue; TRPML subfamily: purple.) *C.FA*, *C. farreri*; H.SA, *H. sapiens*; M.MU, *M. musculus*; G.GA, *G.gallus*; X.TR, *X. tropicalis*; D.RE, *D. rerio*; S.PU, *S. purpuratus*; A.JA, *A. japonicus*; C.IN, *C. intestinalis*; O.BI, *O. bimaculoides*; A.CA, *A. californica*; L.AN, *L. anatina*; D.ME, *D. melanogaster*; C.EL, *C. elegans*; C.GI, *C. gigas*.

A phylogenetic tree was constructed with full-length amino acid sequences of *C.FA* TRPs and other species. As shown in Figure 3, the TRP family is clearly divided into seven branches, which represent seven TRP subfamilies. Each TRP subfamily is clustered according to the evolutionary status of the species. In each subfamily, the TRP members of vertebrates and invertebrates are clustered separately, and the same TRP subfamily of scallop and oyster were first clustered. The support of the evolutionary tree is basically above 70, which shows that the clustering results of the phylogenetic tree are reliable.

The red branch represented the TRPM subfamily. *C.FA* TRPM1 and *C.FA* TRPM3 gathered with TRPM1, TRPM3, TRPM6 and TRPM7 of vertebrate. *C.FA* TRPM2a and *C.FA* TRPM2b clustered with the other four TRPM members of vertebrates TRPM2, TRPM4, TRPM5, and TRPM8. The orange branch was the TRPC subfamily. *C.FA* TRPC-like clustered with TRPC3, TRPC7, and TRPC6 of vertebrates. *C.FA* TRPC-γ clustered with TRPC1, TRPC4, and TRPC5 of vertebrates. Another two *C.FA* TRPC members (*C.FA* TRPC α1 and α) together with other TRPCs of invertebrate clustered outside of TRPCs of vertebrate. The yellow branch represented the TRPA subfamily. Two *C.FA TRPA* genes were presented in this branch. *C.FA TRPA* was more close to TRPA1 of vertebrate than *C.FA* TRPA1-homolog which was at the periphery of the entire TRPA branches. The light blue branch was the TRPV subfamily. Four *C.FA TRPV* genes first clustered with other TRPVs of invertebrates, and then grouped together with TRPV1–6 of vertebrates. Only one TRP member of *C. farreri* was presented in the TRPP subfamily (dark blue branch), TRPN subfamily (green branch), and TRPML subfamily (purple branch). In addition, the numbers of five subfamilies including TRPC, TRPM, TRPV, and TRPML obviously increased in vertebrates, which indicated these TRP subfamilies expanded during evolution. The TRPN subfamily mainly occurred in invertebrates and some fishes, but was not detected in *H. sapiens* and *M. musculus.*

### 2.3. Spatiotemporal Expressions of *C.FA* TRP Genes

**Figure 4:**
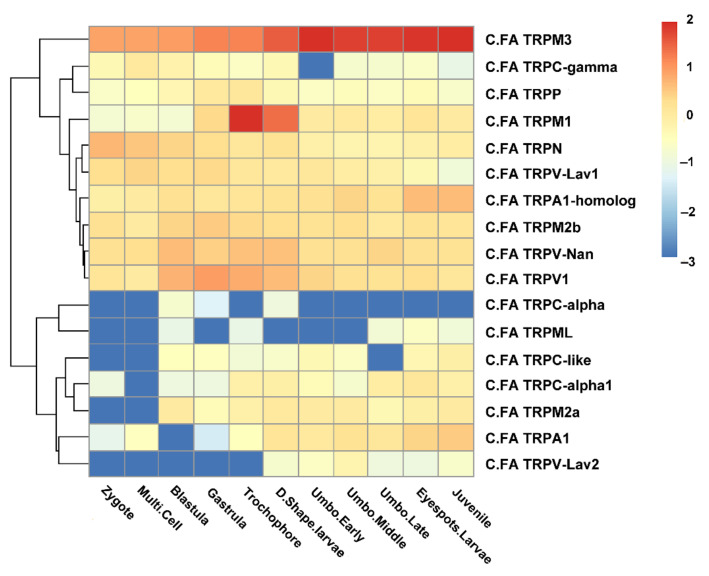
A heat map summarizing the expression of *C.FA TRP* genes during different development stages based on the Log_10_RPKM.

**Figure 5:**
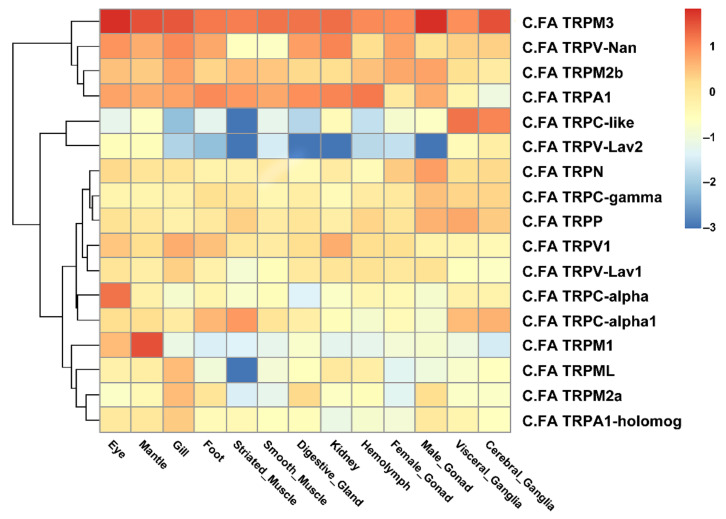
A heat map summarizing the expression of *C.FA TRP* genes in different adult tissues based on the Log_10_RPKM.

RNA-seq datasets for different developmental stages and adult tissues of *C. farreri* were used to detect the spatiotemporal expression profiles of *C.FA TRP*s (Figure 4 and Figure 5). During different developmental stages, the expression pattern of *C.FA TRPs* was obviously divided into two groups. One group consisted of 10 *C.FA TRPs* which continuously expressed during developmental periods. Another group consisted of seven *C.FA TRPs* with a low expression level during developmental periods. In particular, the expression of *C.FA TRPM3* continuously increased during developmental stages and was highest among all *C.FA TRPs*. *C.FA TRPM1* is highly expressed in trochophore and D-shaped larvae stages. The expression of *C.FA TRPN* and *C.FA TRPV-Lav1* were high in early developmental periods and then gradually decreased from gastrula stage. *C.FA TRPA1-homolog* continuously expressed during developmental stages and reached the peak at eyespots larvae stage. *C.FA TRPM2b, *C.FA* TRPV-Nan* and *C.FA TRPV1* highly expressed at blastula, gastrula, trochophore and D-shaped larvae stages. For those 7 *C.FA TRPs* with low expression level, *C.FA TRPC-*α and *C.FA TRPML* almost did not express during development periods. *C.FA TRPC-like*, *C.FA TRPC-*α*1*, *C.FA TRPM2a, C.FA TRPA1*, and *C.FA TRPV-Lav2* did not express or weakly expressed at early developmental stages, but expressed during the middle and late developmental stages.

In adult tissues of the scallops, the expression pattern of *C.FA TRPs* was obviously divided into three groups. The first group consisted of 4 *C.FA TRPs* with a high expression level in almost all tissues (RPKM > 10). The second group consisted of 11 *C.FA TRPs* which expressed with a middle level in most tissues (1 < RPKM < 10). The third group consisted of 2 *C.FA TRPs* with a low expression level in most tissues (RPKM < 1). In particular, *C.FA TRPM3* expressed highly in all tissues. In addition to striated muscle and smooth muscle, *C.FA TRPV-Nan* expressed highly in other tissues. Both *C.FA TRPM2b* and *C.FA TRPA1* expressed highly in other tissues besides the ganglia. *C.FA TRPN* and *C.FA TRPC-γ* expressed highly in the male gonad. The expression level of *C.FA TRPP* was higher in male gonad and ganglia than other tissues. *C.FA TRPV1* mainly expressed in gill and kidney. *C.FA TRPC-α* expressed highly in eye, and *C.FA TRPC-α1* expressed highly in striated muscle, foot and ganglia. The expression of *C.FA TRPM1* was highest in mantle than other tissues. *C.FA TRPML*, *C.FA TRPM2a* and *C.FA TRPA1-homolog* hardly expressed except in the gill. For those 2 *C.FA TRPs* with low expression level, *C.FA TRPC-like* and *C.FA TRPV-Lav2* almost did not express in gill, foot, muscle, digestive gland and hemolymph, while, *C.FA TRPC-like* showed specific high expression in cerebral ganglia and visceral ganglia (RPKM > 10).

### 2.4. Expression of *C.FA* TRP Genes in Response to Heat Stress

**Figure 6:**
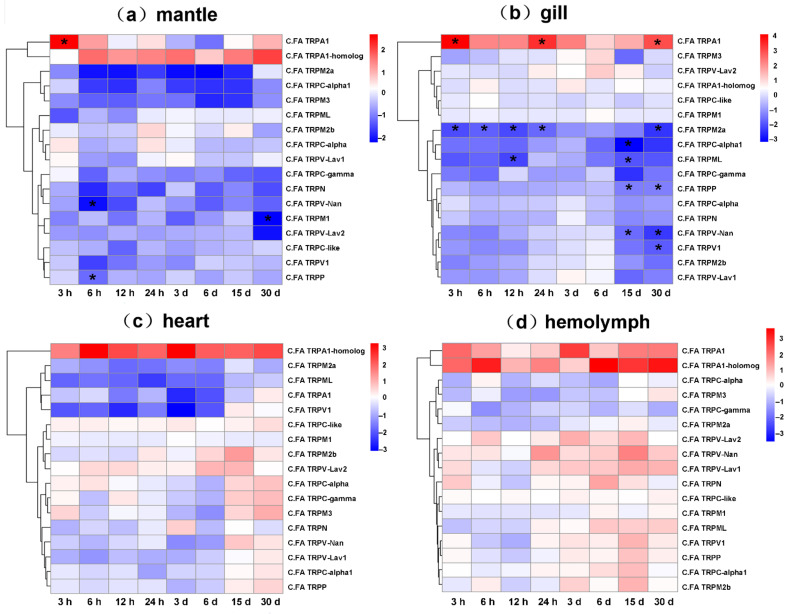
Heatmaps of *C.FA TRP* genes expression under high temperature stress along different time points in mantle (**a**), gill (**b**), heart (**c**) and hemolymph (**d**). The heatmaps were drawn based on log_2_FC values. * indicates significant difference (|log_2_FC| > 1 and *p* < 0.05).

To examine the expression patterns of *C.FA TRP* genes in response to heat stress, RNA-seq datasets of four tissues from *C. farreri* under heat stress were used for analysis. Under heat stress, *C.FA TRPA1* up-regulated in mantle, gill and hemolymph, and *C.FA TRPA1-homolog* up-regulated in mantle, heart and hemolymph (Figure 6 and Table S4), which indicated *C.FA* TRPA subfamily was the thermoTRP channel of scallop. In particular, *C.FA TRPA1* significantly up-regulated (fold change: 6.36, *P* < 0.05) at 3-h post heat stress and *C.FA TRPA1-homolog* started to up-regulate at 6 h and remained up-regulated during the heat stress process in mantle (Figure 6a). The expression of *C.FA TRPA1* was significantly increased at 3 h (fold change: 20.15, *p* < 0.05), 24 h (fold change: 12.27, *p* < 0.05), and 30 days (fold change: 8.94, *p* < 0.05) in gill (Figure 6b). In addition, the expressions of *C.FA TRPV-Nan*, *C.FA TRPP* and *C.FA TRPM1* were significantly down-regulated at 6 h (fold change: −0.21, *p* < 0.05), 6 h (fold change: −0.41, *p* < 0.05), and 30 days (fold change: −0.20, *p* < 0.05), respectively, in mantle (Figure 6a). *C.FA TRPM2a*, *C.FA TRPC-α1*, *C.FA TRPML*, *C.FA TRPP*, *C.FA TRPV-Nan* and *C.FA TRPV1* were significantly down-regulated at some time points under heat stress in gill (Figure 6b). In heart and hemolymph, the expression of *C.FA TRP* genes showed slight fluctuations but were not significantly different with the control under heat stress (Figure 6c,d). *C.FA TRPA1-homolog* showed a trend of high expression in heart. *C.FA TRPA1* and *C.FA TRPA1-homolog* showed a trend of high expression in hemolymph.

## 3. Discussion

The TRP channel is an important cation channel and is widely present in the animal kingdom [46]. When TRP channel is activated, it can transport many cations including Ca^2+^ across the membrane, and participate in the transmission of various sensations such as sight [47], heat [35,36,48], hearing, touch, and osmotic pressure process [49]. In recent years, with the deeper research on the function of TRP family genes, more and more evidence shows that TRP channels are also involved in many physiological process such as embryonic development, blood pressure regulation, intestinal peristalsis, body fluid balance, cell growth and apoptosis, and tumor growth [1,22,24,25]. In this study, we identified a complete set of TRP channels in the genomes of the bivalve *C. farreri* and analyzed the protein structure and phylogenetic relationships. Then, the expression profile was assessed during developmental stages in adult tissues and under heat stress. The results provided insights into the molecular evolution and functional diversity of the TRP channel family.

Through genome-wide screening, 17 TRP family genes were identified in *C. farreri*, which belonged to 7 TRP subfamilies. The number of members of each TRP subfamily in invertebrates was quite different from that in vertebrates. During the evolution of the TRP family, the gene expansion of the five subfamilies TRPC, TRPM, TRPV, TRPP and TRPML was obvious in vertebrates comparing to invertebrates. *D.METRPN* had homologous genes in Lophotrochozoa (such as *C. farreri* and *C. gigas*), while was deleted in mammals (such as *H. sapiens* and *M. musculus)*. In the TRPM subfamily, TRPM2 had multiple copies in *C. farreri* (*C.FA TRPM2a* and *C.FA TRPM2b*), and both of them had ADPRase domains at the C-terminus. It can be speculated that the protein kinase function was distributed between two paralogs during evolution. In the subfamily TRPC, four *C.FA* TRPCs were identified while six to eight TRPCs were found in vertebrates. During the evolution of the TRP family, TRPC2 participated in the transmission of external hormone information from vomeronasal organs in *M. musculus*, while TRPC2 was a pseudogene in *H. sapiens* [16]. These expansion genes should play diverse functions during the evolution process, which was consistent with the existence of TRP family genes in almost all mammalian tissues, as an important bioreceptor involved in many physiological process of the organism [1,2,3,4].

Similar to other TRP proteins of vertebrates and invertebrates, all *C.FA* TRP proteins had six transmembrane domains. In *C.FA* TRPC, *C.FA* TRPA, *C.FA* TRPN and *C.FA* TRPV, there were varying numbers of ANK domains. Like the *D.RE TRPN* and *D.ME TRPN*, *C.FA TRPN* contained 28 ANK domains. ANK domains of *D.RE TRPN* and *D.ME TRPN* were proved to play important roles in the process of perceiving mechanical stimuli [50,51]. The proteins of the two subfamilies, *C.FA TRPP* and *C.FA TRPML*, contained a very large extracellular loop between the first and second transmembrane regions, which was consistent with previous research findings on TRPP and TRPML subfamilies in other organisms [11,20]. The amino acid sequence of TRP family proteins had a sequence identity of 21.70–79.95% with other invertebrate TRP proteins. It can be seen from the results that both the gene structure and protein sequence characteristics showed the conservation of *C.FA TRP* genes compared with other species, and the structural differences among different TRP families may be closely related to their functional differences.

By phylogenetic analysis, the entire evolutionary tree was divided into seven branches, corresponding to seven TRP subfamilies, similar to many published TRP family phylogenetic trees [46]. In *C. farreri* and *C. gigas*, TRP family genes showed a closer relationship, and clustered together with other Lophotrochozoa, which was consistent with the evolutionary relationship of species. The phylogenetic relationship between TRPP and TRPML subfamilies was close, and the support degree reached 97, which was a sister subfamily and a relatively primitive branch in the evolutionary tree. The TRPP subfamily may be the most ancient, as members of this subfamily extend from yeast to mammals [15]. After that, TRPV, TRPN and TRPA clustered as one branch, and TRPM and TRPC clustered as another branch. We hypothesized that as species evolved, their range of existence expanded and environmental factors became more complex, so did the perceptual physiological functions of organisms. To adapt to the complex environment, the species evolved different numbers and functions of the TRP subfamily.

Previous studies have shown that TRP channels played important roles in the development of embryos. For example, during embryonic development, vertebrate asymmetry is closely related to the TRPP2 channel [52]. According to the expression of the TRP family genes of the scallop during developmental stages of *C. farreri*, we found that the expression of *C.FA TRPM3* increased gradually with the development of scallop larvae (In zygote, RPKM = 10, and in juvenile, RPKM = 118). Additionally, *C.EL TRPM* was found to be very important for the post-embryonic mitotic cell divisions of the gonadal precursor cells [32]. We believed that *C.FA TRPM3* played an important role in the development of scallop larvae as an important ion channel. During trochophore and D-shaped larvae stages, scallop larvae swim freely in the water through cilia on the body surface, and the larvae form a primary shell during the D-shaped larvae period. We found that *C.FA TRPM1* was highly expressed during these two periods (RPKM = 114, and RPKM = 34), which indicated *C.FA TRPM1* might play an important role in the proprioception of larvae and the formation of primary shell during these two periods.

As an important non-selective cation channel on the cell membrane, the TRP channel is widely distributed in various tissues and/or Dadongans of organisms. Most TRP channels have high selectivity and permeability for Ca^2+^ which plays an important role in the process of cell growth, proliferation and apoptosis [1,21,22,23,24,25]. According to the expression of TRP family genes in 13 tissues of adult scallops, *C.FA TRPM3* expressed especially with a high level in all tissues, which indicated *C.FA TRPM3* played important roles for adult scallops as a cation channel. Particularly, the RPKM value of *C.FA TRPM3* in scallop male gonad reached 83 which suggested that *C.FA TRPM3* might have a similar role to *C.EL TRPM-gon2* in gonadal precursor cell division [32]. In addition, *C.FA TRPM1* highly expressed in mantle (RPKM = 48), suggesting that it played an important role in the process of mantle tentacles perceiving the external environment. The *C.EL TRPC-trp3* gene expressed in *C. elegans* sperm and mediated Ca^2+^ influx and affected the interaction of sperm and egg membrane, resulting in fertilization. Mutation of *C.EL TRPC-trp3* caused *C. elegans* infertility [53]. Nevertheless, *C.FA TRPC* family members showed weak expression in gonads of scallop, which indicated that the TRP family of scallops and *C. elegans* involved in the function of sperm Ca^2+^ cation channels may be different. *C.FA TRPC-alpha* showed a specific high expression pattern in the eyes of scallops (RPKM = 22). TRPC was first discovered in the *D. melanogaster* light-sensing conduction system and was proven to play an important role in *D. melanogaster* light-sensing conduction [5,6,7]. Therefore, we speculated that similar to *D. melanogaster*, *C.FA TRPC-α* was an important receptor in the light stimulus response of scallop.

Animals have evolved sophisticated physiological systems for sensing ambient temperature, since changes in environmental temperatures affect various biological process [54]. ThermoTRP channels have been proven to serve as thermal sensors in diverse animal species over the past several years [11,33,54]. In mammals, the heat-sensitive TRP family genes include TRPV (TRPV1, TRPV2, TRPV3, TRPV4) and TRPM (TRPM2, TRM3, TRPM4, TRPM5) [33,55], and cold-sensitive TRP family is the TRPA subfamily. However, studies in invertebrates have shown that TRPA subfamily genes serve as thermal sensors. For example, in *D. melanogaster*, the TRPA subfamily members *D.ME TRPA-Pyx*, *D.ME TRPA-Pain* and *D.ME TRPA1* can be activated within a certain temperature range, and mediate *D. melanogaster* to sense the surrounding temperature and respond [38,39]. *D.ME TRPA-Pyx* can be directly activated when the temperature is ≥40 °C, so that *D. melanogaster* can avoid high temperature damage. *D.ME TRPA-Pain* and *D.ME TRPA1* play an important role in the process of *D. melanogaster* avoiding harmful high temperature. Additionally, *D.ME TRPA1* is also a necessary gene for *D. melanogaster* larvae to choose a temperature preference of 18 °C. In recent research, three TRP genes (*CgiTRPC3.6*, *CgiTRPC3.7* and *CgiTRPV4*.7) were found to be related to thermal regulation toward heat tolerance in *C. gigas* [56]. In the four tested tissues of *C. farreri* under heat stress, the up-regulated genes belonged to TRPA family (*C.FA TRPA1* and *C.FA TRPA1-homolog*), which is consistent with the previous findings of *D. melanogaster*. In the mantle, *C.FA TRPA1* was significantly up-regulated at 3 h under high temperature stress, and *C.FA TRPA1-homolog* was up-regulated at each time point except 3 h. In the gill, *C.FA TRPA1* was significantly up-regulated at 3 h, 24 h and 30 d while *C.FA TRPA1-homolog* did not change significantly. In the heart, under high temperature stress, *C.FA TRPA1-homolog* upregulated expression at all time points while *C.FA TRPA1* down-regulated. In the hemolymph, *C.FA TRPA1* and *C.FA TRPA1-homolog* up-regulated at each time point under heat stress.

The molecular mechanism of temperature activated TRP channel is a hot research topic. The corresponding temperature of TRPV1 in mammals is >40 °C, and marks peripheral neurons responsible for detecting noxious heat. However, related studies have found that both squirrels (*Ictidomys tridecemlineatus*) and camels *(Camelus ferus*) express TRPV1 channels with dramatic decreases in thermosensitivity in the physiologically relevant range, and by low-cost replacing single conserved amino acid, squirrel and camel TRPV1 can regain heat sensitivity [57]. Some recent studies using unbiased random mutagenesis and cysteine accessibility pointed out that the pore domain (PD) was a structure specifically related to temperature activation [58,59,60,61]. The PD of TRPM8 of animals living in cold environments (such as *Aptenodytes forsteri*) was less hydrophobic than that of animals living in hot environments (such as *L. africana*). The TRPM8 cold sensitivity of *A. forsteri* was significantly lower than that of *L. africana*. It was speculated that animals adjusted overall hydrophobicity of the amino acids in the TRPM8 pore region to better adapt to the environmental temperature during evolution [62]. Based on high-throughput mutagenesis, it has been shown that TRPV1 heat activation was specifically sensitive to strong decreases in amino acid hydrophobicity in mouse [63]. In *C. farreri*, we found that the GRAVY (Grand average of hydropathicity) of *C.FA TRPA1* PD was greater in the entire *C.FA TRP* genes (Table 2), which is inconsistent with the discovery of the TRPV1 in mouse. This may be due to differences in gene structure and species.

**Figure 7:**
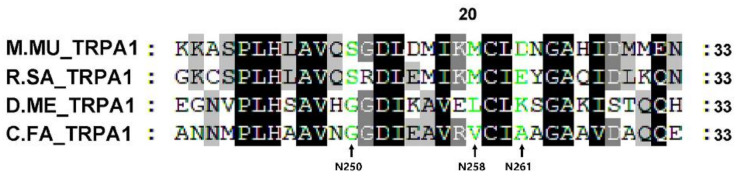
Multi-sequence comparison of the ANK6 in TRPA. The black background represents the same amino acid, the gray background represents similar amino acid, and the green represents the amino acid at the mutation site. M.M, *M.musculus;* R.SA, *Pantherophis obsoletus lindheimeri;* D.ME, *D. melanogaster*; C.FA, *C. farreri*.

The existence of different TRPA1 isoforms and studies on the TRPA1 and TRPV1 chimera methods indicated that a large N-terminal region was involved in the regulation of thermal sensitivity [64,65,66,67]. A prominent feature of TRP channels is that different members of the relatively homologous TRP channel may have opposite thermal sensitivities. For example, TRPA1 of *H. sapiens*, *M. musculus* and *C. elegans* were cold activated, while TRPA1 of rattlesnake, rat snake and fly were heat activated [68]. The directional activation of TRPA1 channel temperature in mouse was found to be reversed by a single-point mutation of Ankyrin Repeat Six (cold activation to hot activation) [69]. In this study, the amino acids at several key positions of Ankyrin Repeat Six of *C.FA TRPA1* (G250) were consistent with that of fly (G250), but were inconsistent with mouse (S250) (Figure 7), which further proved that *C.FA* TRPA1 is a heat-sensitive TRP family channel. 

Comparing the gene expression of the TRP family in the four tissues under heat stress, we found that some genes showed down-regulated expression, such as *C.FA* *TRPM2a*, *C.FA* *TRPV1*, *C.FA* *TRPML*, and *C.FA* *TRPP*. In *C. elegans*, *C.EL* *TRPV* has been shown to be related to the transmission of mechanical and osmotic pressure stimuli, *C.EL* *TRPPs* are related to mating behavior, and *C.EL* *TRPM*s are related to bowel rhythm and defecation [12]. In addition, high temperature stress has been proven to seriously affect the metabolism level of shellfish, and inhibit the function of mitochondria and the ability of cell adsorption and phagocytosis, which further affected the physiological function of scallop tissues and eventually led to death [70,71]. These down-regulated expression of TRP channels of scallops indicated that a variety of sensory physiological functions were affected under high temperature stress, which need further experiments to verify the functions of TRP channels in scallops.

## 4. Materials and Methods

### 4.1. Genome-Wide Identification and Sequence Analysis of TRP Genes in C. farreri

To identify the *TRP* genes, the transcriptome and whole genome sequence databases of Zhikong scallop [72] were searched using available TRP protein sequences of invertebrates *Strongylocentrotus purpuratus*, *Apostichopus japonicus*, *C. intestinalis*, *Crassostrea gigas*, *Octopus bimaculoides*, *Aplysia californica*, *Lingula anatina*, *D.melanogaste*, and *C. elegans*, and the vertebrates *H. sapiens*, *M. musculus*, *Gallus*, *X. tropicalis*, and *D. rerio* in the databases of NCBI (http://www.ncbi.nlm.nih.gov accessed on 1 September 2021) and Uniprot (https://www.uniprot.org/ accessed on 1 September 2021) using TBLASTN with an e-value of 1E-05. BLASTN was then used to align the predicted cDNA sequences with the whole-genome sequences to obtain their genomic structures. ORF Finder (https://www.ncbi.nlm.nih.gov/orffinder/ accessed on 1 September 2021) and DNAstar (version 4.05) (DNASTAR, Madison, WI, USA) were used to predict amino acid sequences. To further confirm the predicted amino acid sequences, BLASTP was conducted against the NCBI non-redundant protein sequence database. The translated sequences were submitted to the SMART program (http://smart.embl-heidelberg.de/ accessed on 1 September 2021) for identification of the signal peptide and other conserved TRP domains. The GRAVY (Grand average of hydropathicity) of pore domain, putative isoelectric point (pI) and molecular weight (Mw) were computed using the Compute pl/Mw tool (https://www.expasy.org/ accessed on 1 September 2021). Geneious7.0.6 (Biomatters Ltd., Auckland, New Zealand) was used to predict secondary structure (http://www.geneious.com/ accessed on 1 September 2021). The protein structures of all the identified TRP proteins were drawn with IBS1.0.3 software (CUCKOO Workgroup, Guangzhou, China) [73].

### 4.2. Multiple Alignment and Phylogenetic Analysis

The TRP proteins from *C. farreri* and other selected species, including *H. sapiens, M. musculus, G. gallus, X. tropicalis, D. rerio, S. purpuratus, A. japonicus, C. intestinalis, C. gigas, O. bimaculoides, A. californica, L. anatina, D. melanogaster,* and *C. elegans*, were chosen for phylogenetic analysis. The TRP amino acid sequences from these species were retrieved from the NCBI and Uniprot databases (Table S1). Multi-sequence alignment of TRP domain was performed using ClustalW [74], and then was edited by Genedoc software (Pittsburgh Supercomputing Center, Pittsburgh, PA, USA) [75]. The NCBI database (https://blast.ncbi.nlm.nih.gov/Blast.cgi accessed on 1 September 2021) was used to compare the identity of TRP protein sequences. The phylogenetic tree was constructed using the neighbor-joining method with MEGA 7 [76]. Bootstrap method was used for phylogeny test with 1000 replications. The bootstrap values were added in all branches of phylogenetic tree.

### 4.3. Spatiotemporal Expression Profiles of C.FA TRP Genes

For expression analysis, the RPKM (reads per kilo per million reads) values of each *TRP* gene were retrieved from the published RNA-seq datasets of *C. farreri* including various developmental stages (zygote, multicell, blastula, gastrula, trochophore, D-shaped larvae, early umbo, middle umbo, post umbo, eyespots larvae, juvenile) (Table S2) and adult tissues (eye, mantle, gill, foot, striated muscle, smooth muscle, digestive gland, kidney, hemolymph, female gonad, male gonad, visceral ganglia, cerebral ganglia) (Table S3). The RPKM values were Log_10_ transformed and subsequently used to draw a heat map with custom R scripts.

### 4.4. Expression of C.FA TRP Genes Under Heat Stress

Healthy Zhikong scallops (*n* = 100), of which the average shell height was 54.95 mm (±4.91), were collected from the scallop farming area in Qingdao (Shangdong Province, China) in October 2018. The scallops were transported to the laboratory and acclimated for one week prior to the high temperature stress experiments. During this period, scallops were kept in filtered and aerated seawater at salinity 25 ppt, pH 8.0, temperature 20 °C, which is consistent with the sampling environment. Scallops were fed two times per day with *Nitzschia closterium* (1.0 × 10^5^ cells/scallop) and the seawater was replaced daily. After acclimation, nine scallops were randomly sampled before challenging experiment as the control group (temperature 20 °C), and their mantle, gill, heart, and hemolymph were separated and immediately frozen in liquid nitrogen. The remaining scallops were randomly divided into three groups and transferred to seawater at 27 °C which was close to the maximum temperature at sampling location. At 3-h, 6-h, 12-h, 24-h, 3-d, 6-d, 15-d and 30-d post heat stress, nine scallops were randomly dissected and the four tissues, same with control, were collected and frozen for subsequent RNA extraction.

Total RNA was isolated from the mantle, gill, heart, and hemolymph of three individuals at each sampled time point. RNA-seq libraries were constructed according to the standard illumine protocols and sequenced by Illumina Hiseq 2000 platform (Illumina, San Diego, California, USA). RNA-seq reads were then mapped to the *C. farreri* genome using Tophat (ver 2.0.9) (University of Maryland, City of College Park, MD, USA), and expression of all *C.FA TRP* genes were calculated with the form of TPM (Transcripts Per Million) (Table S4). Fold change (FC) was calculated as log_2_FC between each heat stress time point and control group. Differentially expressed genes were identified using edgeR package with statistically significant cutoff of |log_2_FC| > 1 and *p* value < 0.05. The log_2_FC values were used to draw a heat map with custom R scripts.

## 5. Conclusions

In summary, a total of 17 *TRP* genes were identified from genomes of the Zhikong scallop. All *C.FA* TRPs are highly conserved in its sequence and structural features. Expression profiles of *C.FA* *TRPs* during developmental stages and in adult tissues provided preliminary functional implications for bivalve *TRP* genes. Some *C.FA* *TRP* genes participate in the early embryonic development and sensory process of adult tissues. *C.FA* *TRPA1* and *C.FA* *TRPA1-homolog* upregulated significantly under heat stress which indicated TRPA should be the thermoTRP channel of scallop. Our study provides the first genome-wide investigation of TRP channels in bivalves and will assist in better understanding of TRP function and evolution in Mollusca.

## Figures and Tables

**Table 1:** Statistical table of gene members of TRP subfamily in different species.

Species	TRPC	TRPM	TRPV	TRPA	TRPP	TRPML	TRPN	Total	References
*Homo sapiens*	6	8	6	1	3	3	0	27	[11]
*Mus musculus*	7	8	6	1	3	3	0	28	[11]
*Danio rerio*	8	6	4	2	4	2	1	27	[10]
*Ciona intestinalis*	8	2	2	4	1	9	1	27	[9]
*Chlamys farreri*	4	4	4	2	1	1	1	17	in this article
*Drosophila melanogaste*	3	1	2	4	1	1	1	13	[8]
*Caenorhabditis elegans*	3	4	5	2	1	1	1	17	[12]

**Table 2:** Sequence characteristics of *TRP* gene family of *C. farreri*.

Gene Name	Gene ID	cDNA Length (bp)	ORF Length (bp)	Exons No.	Introns No.	Amino Acid No.	Molecular Weight (kDa)	Theoretical pI	AlpHa No.	Beta No.	Colis No.	Turns No.	GRAVY of PD
*CfTRPC-α*	scaffold62023.33.1	3103	2847	15	14	948	109.29	5.42	44	47	63	68	0.601
*CfTRPC-α1*	scaffold51977.10.1	3131	2493	13	12	830	96.25	7.3	40	46	53	61	0.612
*CfTRPC-γ*	scaffold17795.40.2	2721	2592	10	9	863	99.15	6.85	40	44	57	62	0.355
*CfTRPC-like*	scaffold53059.6	3295	2724	10	9	907	104.40	7.95	36	46	69	68	0.526
*CfTRPM2a*	scaffold28131.6	4248	4248	27	26	1415	161.45	8.48	71	76	104	103	0.656
*CfTRPM2b*	scaffold64377.34	4875	4875	34	33	1624	184.87	5.98	74	85	119	116	0.764
*CfTRPM1*	scaffold1.4.1	5847	3072	17	16	1023	116.70	6.75	54	52	61	61	0.56
*CfTRPM3*	scaffold52123.6.1	5373	5052	25	24	1683	191.64	6.67	83	87	113	112	0.572
*CfTRPA1*	scaffold61233.39	3453	3453	26	25	1150	128.73	7.05	62	63	64	70	1.184
*CfTRPA1-homolog*	scaffold63953.15	5170	3564	30	29	1187	133.42	6.5	63	60	68	65	0.994
*CfTRPN*	scaffold29423.13	4716	4716	26	25	1571	172.82	7.87	82	78	99	86	0.745
*CfTRPV-Nan*	scaffold63985.6	3322	1980	12	11	659	75.07	6.03	22	32	40	43	0.503
*CfTRPV1*	scaffold57527.16	2487	2487	17	16	828	95.56	8.71	43	45	47	55	0.623
*CfTRPV-Lav1*	scaffold24983.12	3473	3072	16	15	1023	116.59	8.86	50	52	67	68	0.75
*CfTRPV-Lav2*	scaffold64873.6	2283	1953	1	0	650	73.75	7.57	33	36	45	37	0.634
*CfTRPP*	scaffold64453.6.1	3767	2571	15	14	856	98.04	5.36	32	41	73	70	−0.265
*CfTRPML*	scaffold5673.1	2257	1614	13	12	537	60.54	6.37	28	36	27	35	1.162

**Table 3:** Percentage of Identity(I) of *CfTRPs* with selected TRP proteins in other species.

Gene	*H. sapiens*	*M. musculus*	*X. tropicalis*	*D. rerio*	*C. gigas*	*D. melanogaster*	*C. elegans*
*CfTRPC-α*	29.05%	29.05%	28.22%	28.50%	48.07%	32.92%	26.98%
*CfTRPC-α1*	32.82%	32.82%	31.75%	31.28%	69.28%	34.21%	26.37%
*CfTRPC-γ*	40.52%	40.39%	40.62%	40.03%	77.74%	57.09%	52.68%
*CfTRPC-like*	35.93%	35.80%	35.88%	35.34%	66.15%	40.53%	50.41%
*CfTRPM2a*	36.70%	37.04%	32.18%	31.91%	45.35%	34.33%	27.42%
*CfTRPM2b*	31.00%	31.50%	31.47%	31.80%	46.90%	32.06%	34.53%
*CfTRPM1*	41.77%	41.55%	38.29%	38.99%	53.39%	39.16%	30.95%
*CfTRPM3*	49.45%	49.69%	50.29%	47.41%	66.51%	51.36%	54.58%
*CfTRPA1*	37.25%	36.80%	36.62%	36.56%	55.41%	40.56%	24.39%
*CfTRPA1-homolog*	27.72%	27.65%	24.63%	26.11%	25.57%	26.18%	23.62%
*CfTRPN*	/	/	/	57.13%	79.95%	50.55%	49.86%
*CfTRPV-Nan*	27.47%	26.93%	27.74%	27.29%	69.54%	47.57%	39.69%
*CfTRPV1*	19.80%	19.69%	20.23%	19.12%	56.05%	28.12%	21.70%
*CfTRPV-Lav1*	28.62%	28.45%	29.61%	25.87%	57.25%	45.91%	45.64%
*CfTRPV-Lav2*	25.47%	25.66%	25.71%	23.46%	53.82%	43.07%	42.00%
*CfTRPP*	50.55%	50.19%	50.06%	52.53%	76.98%	37.88%	37.03%
*CfTRPML*	26.59%	25.95%	26.93%	26.10%	28.87%	27.41%	23.40%

## Data Availability

All the data used in this study have been provided in the main text and Supplementary Materials.

## Supplementary Materials

The following are available online at https://www.mdpi.com/article/10.3390/ijms222011075/s1.
